# The Influence of Mind upon Body

**Published:** 1896-02-15

**Authors:** 


### The Influence of Mind upon Body.

It is matter for no small conceit that man, even though he be a little lower than the angels, at least is higher than the brutes. It is, however, far from a true conception of the facts to look upon man's organ of mind as a something separate, a final corner-stone added to an already perfect organisation. Just as in the earlier stages in animal development the rudimentary brain carried on work which in more lowly creatures was done by scattered masses of nervous tissue, just, indeed, as these lowly brains might be looked upon as aggregations of nervous tissue, placed where they are for reasons of convenience, if one may so say, but still having their old connection with each portion of the body, so, in regard to our higher brains, it will not do to think that even the highest portion of the organ of mind, the very substratum of consciousness, is a something separate superadded to an independent nervous system. No. The mind, even in its highest exercise, cannot sever itself from the influence of the body, and the very cells in the cortex, whose consentaneous actions are the bases of our thoughts, and whose interlinking is the machinery of our minds, are also interlinked with those controlling the lowest and most animal functions of the body.

The influence of body upon mind is generally admitted, and historians tell us how at critical periods the world's progress has been influenced by the small ailments of generals and statesmen; but the persistent influence of mind upon body is not so generally recognised. Everybody knows that a great shock will stop digestion, or that a great terror will set up diarrhœa; these, however, are exceptional affairs. It is the constant all-pervading influence of the cerebral cortex on the daily details of organic life which is apt to be overlooked in arranging lines of treatment for what are considered purely bodily ailments. In a masterly address, recently delivered before the Royal Medical Society, at Edinburgh, Dr. Clouston has pointedly drawn attention to this, and to the great importance of studying the mental state as well as the physical condition. The very exactitude of many of the means of diagnosis now at the disposal of the physician tempts him to put out of consideration what is incapable of being touched and measured. As Dr. Clouston says, if some people want to imply that a patient's symptoms are unimportant they call them "nervous"; if they want to ticket them as unworthy of consideration altogether they call them "mental"; and if they want to brand them as quite absurd and out of the pale of human sympathy or medical effort, they call them "hysterical." If this is so in regard to definite symptoms of which complaints are made, how much more are mental or nervous conditions, which occur alongside of obvious organic disease, likely to be ignored. Yet they are of the greatest importance, not only because they influence the progress of the disease, but because, as Dr. Clouston has shown, they may actually be the prime cause of its occurrence. That condition of harmonious co-operation of all parts of the body which we call health is a matter of brain quite as much as of organs. "The evidence that the brain cortex regulates absorption, secretion, vascular tone, and the anabolic and katabolic processes in the cells of the tissues, may now be regarded as complete." The bearing of this upon matters of disease and therapeutics is plain. It would appear as if certain persons had as their great protective and prophylactic against disease a sound and well-working mind and brain cortex. When well in mind they are sound in body; when disturbed in mind they fall victims to their diathesis. Death takes place with most men not because disease is overmastering, for they may have got safely through more serious attacks many times before, but because the resistive power is lessened; and this resistive power is a question not merely of liver or of kidneys, but of all parts of the economy, including the brain cortex. Again this is an extreme instance, but none the less does it appear certain that in even minor ailments mental influences are effective either for good or evil. It is not only fresh air and sunshine that we seek in health resorts, but an easy mind; while, on the other hand, the indigestions of city life do not, as some would think, all spring from "nips" and overfeeding, but much from worry. A physician must take large views. It is as idle to prescribe for a man's liver without considering his ambitions, as it is to treat a girl's chlorosis without thinking of her love affairs.

